# Using health facility deaths to estimate population causes of neonatal and child mortality in four African countries

**DOI:** 10.1186/s12916-020-01639-1

**Published:** 2020-06-12

**Authors:** Henry D. Kalter, Jamie Perin, Agbessi Amouzou, Gift Kwamdera, Wasilat Adeyinka Adewemimo, Félicitée Nguefack, Abdoulaye-Mamadou Roubanatou, Robert E. Black

**Affiliations:** 1 Institute for International Programs, Johns Hopkins Bloomberg School of Public Health, Baltimore, MD USA; 2 Center for Child and Community Health Research, Department of Pediatrics, Johns Hopkins School of Medicine, Baltimore, MD USA; 3 Queen Elizabeth Central Hospital, Ministry of Health, Blantyre, Malawi; 4 Department of Health Planning, Research & Statistics, Federal Ministry of Health, FCT, Abuja, Nigeria; 5 Department of Pediatrics, Faculty of Medicine and Biomedical Sciences, University of Yaoundé I, Yaoundé, Cameroon; 6 Ministry of Public Health, Niamey, Niger

**Keywords:** Neonatal mortality, Child mortality, Cause-specific mortality fraction, Health facility, Community, Verbal autopsy

## Abstract

**Background:**

Verbal autopsy is the main method used in countries with weak civil registration systems for estimating community causes of neonatal and 1–59-month-old deaths. However, validation studies of verbal autopsy methods are limited and assessment has been dependent on hospital-based studies, with uncertain implications for its validity in community settings. If the distribution of community deaths by cause was similar to that of facility deaths, or could be adjusted according to related demographic factors, then the causes of facility deaths could be used to estimate population causes.

**Methods:**

Causes of neonatal and 1–59-month-old deaths from verbal/social autopsy (VASA) surveys in four African countries were estimated using expert algorithms (EAVA) and physician coding (PCVA). Differences between facility and community deaths in individual causes and cause distributions were examined using chi-square and cause-specific mortality fractions (CSMF) accuracy, respectively. Multinomial logistic regression and random forest models including factors from the VASA studies that are commonly available in Demographic and Health Surveys were built to predict population causes from facility deaths.

**Results:**

Levels of facility and community deaths in the four countries differed for one to four of 10 EAVA or PCVA neonatal causes and zero to three of 12 child causes. CSMF accuracy for facility compared to community deaths in the four countries ranged from 0.74 to 0.87 for neonates and 0.85 to 0.95 for 1–59-month-olds. Crude CSMF accuracy in the prediction models averaged 0.86 to 0.88 for neonates and 0.93 for 1–59-month-olds. Adjusted random forest prediction models increased average CSMF accuracy for neonates to, at most, 0.90, based on small increases in all countries.

**Conclusions:**

There were few differences in facility and community causes of neonatal and 1–59-month-old deaths in the four countries, and it was possible to project the population CSMF from facility deaths with accuracy greater than the validity of verbal autopsy diagnoses. Confirmation of these findings in additional settings would warrant research into how medical causes of deaths in a representative sample of health facilities can be utilized to estimate the population causes of child death.

## Background

Accurate information on the causes of neonatal and child mortality is needed to help prioritize health expenditures and shape effective health policies and programs in developing countries. It is a long-held assumption in the international public health literature that the causes of child deaths in health facilities, mainly hospital, differ from those that occur in the community [1, 2]. These differences are likely to be even more exaggerated at secondary and especially tertiary care hospitals that provide specialized services including neonatal intensive care to which severely ill patients are referred from lower level facilities. At the same time, evidence shows that half or more of child deaths in most developing countries occur at home [2–4], with many of these children having received no formal health care during the course of their fatal illness [4–8]. Civil registration and vital statistics (CRVS) systems with medical certification of the causes of death in such settings are often weak [9, 10]. Thus, arises the need for verbal autopsy (VA), the widely used and generally accepted best available method for estimating cause of death at the population level in settings with inadequate medical certification, until such time as functioning CRVS systems are put in place.

The VA method involves a structured interview of the child’s main caregiver to identify the signs and symptoms (hereafter referred to as “illness signs” or “signs”) of the fatal illness, such as fever, rash, difficulty breathing, and loose or liquid stools, from which the cause of death is estimated. There are several methods available to assess cause of death from VA data, the most widely used including physician coding (PCVA) [11], expert algorithms (EAVA) [12], Tariff [13], InterVA [14], and InSilicoVA [15]. The validation of verbal autopsy methods has been by comparison to causes of hospital-based deaths, leaving the question of how well verbal autopsy can ascertain causes of death in the community.

Even if the causes of death in hospital and the community were identical, some concerns would remain. Limited access to health care, especially at the secondary and tertiary levels, is a serious problem in many developing countries, and persons of higher socioeconomic and educational status may be overly represented among those who receive formal health care. These factors, and the hospital experience itself, might affect recognition, recall, and reporting of illness signs. In addition, because hospitalization might alter illness course and so the illness signs present for observation, respondents for community and hospital deaths might witness and report somewhat different signs in association with the same cause of death.

Therein lies the VA paradox, that its validation must rely on hospital deaths for the very reason that VA is needed, because cause of death in the community cannot be established with sufficient accuracy to serve as a standard for testing VA methods. Yet the reliance on hospital data for its validation calls into question VA’s accuracy in assessing cause of death in the community.

A well-selected sample of health facilities, representative of the deaths occurring at all health facilities in a given geographic area, with proper medical diagnosis and certification, could represent the causes of death not only in facilities but also in the community at-large [16]. Moreover, in the case that facility deaths differed from community deaths, they could potentially be modeled using periodically collected census or survey data to predict the causes in community and thus at the population level [17, 18].

But how to test this methodology, when the medical causes of community deaths cannot be determined with sufficient accuracy to even support the validation of verbal autopsy? Again, we must turn to VA for a feasible approach to the problem. If the same VA methodology was used to determine the causes of death both in health facilities and the community, then the causes in these two spheres should be reasonably comparable, short of the potential effect of demographic factors that could be adjusted for.

From 2012 to 2016, we conducted verbal/social autopsy (VASA) studies of neonatal and child deaths in four African countries. We undertook the current analysis to determine how similar or dissimilar were the causes of death that occurred in health facilities and the community; to assess the implications of these findings on the use of VA for the identification of neonatal and child causes of death at the population level; and if causes of community and facility deaths were dissimilar, to determine whether community causes could be estimated from the facility causes with adjustment using a small set of demographic factors.

## Methods

We conducted VASA studies of representative samples of neonatal (0–27 days old) and child (1–59 months old) deaths in Cameroon, Malawi, Niger, and Nigeria. Several publications describe the full verbal autopsy methods and findings of two of these studies [19, 20]. In brief, deaths in each country were identified by a full birth history of all women participating in a household survey of a representative population sample: in eastern Cameroon, the 2010 baseline census of 16,954 households for the Population Services International Community Case Management study in Doume, Nguelemendouka, and Abong-Mbang districts [21]; in central and southern Malawi, the 2011–2012 midline survey of 24,000 households for the Real-time Mortality Monitoring project in Balaka and Salima districts [22]; in Niger, the 2011 National Mortality Survey of 25,024 households [23]; and in Nigeria, the 2013 national Demographic and Health Survey of 38,522 households [24]. The VASA studies sampled one under-5 death from each surveyed household with one or more such deaths, and conducted a VASA interview with each child’s main caregiver using the Population Health Metrics Research Consortium VA-Johns Hopkins University/Institute for International Programs SA questionnaire [25]. The final sample sizes and recall periods from death to interview, respectively, of neonatal and 1–59-month-old deaths with completed VASA interviews were 164 (mean 3.6 years, range 2–6 years) and 635 (3.0, 2–6) in Cameroon, 320 (2.3, 0–4) and 691 (2.5, 0–4) in Malawi, 453 (3.5, 2–5) and 619 (2.7, 2–5) in Niger, and 722 (3.6, 1–6) and 2055 (3.8, 0–6) in Nigeria.

The EAVA and PCVA analysis methods were utilized in each country to identify the proportions of neonatal and 1–59-month deaths from, respectively, 10 and 12 causes. The PCVA analysis was conducted by one well-trained local physician (FN, GK, A-MR, and WAA, respectively, in Cameroon, Malawi, Niger, and Nigeria) employing pre-defined required minimal diagnostic criteria combined with their clinical judgment. The physician completed a death certificate for each case, and the underlying cause appearing on the lowest line of section 1 of the certificate was selected for analysis. The EAVA method utilizes pre-defined expert algorithms based on illness signs and symptoms reported during VA interviews, arranged in a hierarchy that follows ICD-10 rules to the extent possible to select the underlying cause of death [12]. To estimate cause distributions in Malawi, Niger, and Nigeria, survey weights were applied and primary sampling units handled such that all analyses accounted for the multi-stage sampling designs of the platform surveys. Survey weights were not required in Cameroon, where the deaths were identified through a population census of the three study districts.

### Statistical tests

We conducted three analyses. First, for each country, we compared the proportions of deaths from each neonatal and 1–59-month EAVA and PCVA cause of death that occurred in the community and in health facilities. The VASA questionnaire categorized the place of death as “hospital,” “other health provider or facility,” “on route to a health provider or facility,” “home,” or “other.” Health facility deaths included deaths from “hospital” or “other health provider or facility” and were further categorized as “health center,” “health post,” or “private doctor/clinic.” Deaths that occurred at home, on route to a health provider of facility, or other place were grouped as community deaths. “Other health providers” were separately determined to be trained community health workers, nurses, or midwives, and were included in the “other health provider or facility” group only if they were seen at a health facility. A Rao-Scott chi-square or Fisher exact test was used to assess the significance of any differences between the community and health facility cause-specific proportions [26].

Next, we estimated the CSMF accuracy [27] of the overall community/facility cause distributions of neonatal and 1–59-month deaths in each country accounting for the survey design. CSMF accuracy was originally designed to compare a non-reference to a reference standard CSMF, where 0 represents extreme differences and 1 represents no difference [27]. Here, we used this statistic to assess the level of correspondence between two non-reference CSMFs. We approximated 95% confidence intervals for this statistic by bootstrapping based on resampling primary survey sampling units [28].

In addition, we compared the observed population (facility plus community) distribution of the EAVA and PCVA causes of death for neonates and children to their corresponding predicted distributions, using several different methods to predict for causes of community death in a hypothetical scenario where community causes had not been measured and only the causes of facility deaths were available. First, in a “naïve” prediction (projection A), we used the observed cause distribution of facility deaths to represent population distributions, effectively assuming that causes in communities were the same as in facilities. We compared the resulting prediction of population-level cause distributions to the observed population distribution using CSMF accuracy. We also modeled neonatal and 1–59-month causes of death using multinomial logistic regression (MLR) [29] and random forests (RF) [30] to adjust for the potential influence of several factors on the community and health facility causes. We selected adjustment factors based on their availability in common population surveys such as the Demographic and Health Survey (DHS) conducted in many developing countries, to facilitate possible future use of the prediction models by public health officials.

We adjusted for age at death and mother’s education, as well as whether the mother received any antenatal care (for neonates). We estimated these models among deaths occurring in facilities and used the results to predict the causes of death for those occurring in the community, which proportionally combined with the facility causes of death provided a population-level prediction (MLR projection B). Additional factors were considered but not included in this MLR projection due to model instability. We used random forests to allow additional factors to influence projected causes. We built the random forest classification with deaths occurring in facilities based on age at death, mother’s education, child sex, birthplace, and wealth quintile, as well as, for neonates, whether the mother received antenatal care (RF projection C). We predicted the causes of death in the community with this model and combined these with the facility deaths to estimate the population-based cause of death distribution, which we compared to the observed population-based distribution using CSMF accuracy. Lastly, we conducted the random forest analysis utilizing only the predictors included in the multinomial regressions (projection D) and using the same predictors included in the multinomial regressions, plus birthplace (projection E).

We also examined CSMF accuracy by the percent of deaths occurring in facility. Within each study, we assumed a fixed cause distribution among the community and facility deaths, and then varied their weights to determine the population-based CSMF. All analyses were conducted in R version 3.5.0.

## Results

### Comparison of specific causes

Table 1 shows the EAVA and PCVA population cause-specific proportions and 95% confidence intervals of the neonatal and 1–59-month-old deaths in the four countries. Across the four study countries, the percent of neonatal and 1–59-month deaths that occurred in facilities ranged, respectively, from 18.8 to 55.0% and 18.6 to 50.5%. Only in Malawi did facility deaths exceed 50%.

**Table 1:** Cause-specific proportions of neonatal (0–27 days) and child (1–59 months) deaths in the study countries

Cause of death	Cameroon		Malawi		Niger		Nigeria	
	164 neonatal deaths, 635 child deaths		320 neonatal deaths, 691 child deaths		453 neonatal deaths, 619 child deaths		722 neonatal deaths, 2055 child deaths	
	% (95% CI)	% (95% CI)	% (95% CI)	% (95% CI)	% (95% CI)	% (95% CI)	% (95% CI)	% (95% CI)
	EAVA	PCVA	EAVA	PCVA	EAVA	PCVA	EAVA	PCVA
***Neonates***								
**Tetanus**	1.2 (0.2, 3.8)	0.0 (−,^±^ −)	1.4 (0.5, 3.1)	0.0 (−, −)	4.0 (2.2, 6.5)	0.4 (0.1, 1.3)	1.2 (0.6, 2.2)	0.4 (0.1, 0.9)
**Congenital**	3.0 (1.1, 6.5)	0.0 (−, −)	0.3 (0.0, 1.3)	0.0 (−, −)	2.7 (1.3, 4.7)	0.2 (0.0, 0.9)	0.9 (0.3, 2.2)	1.1 (0.2, 3.4)
**BA/BI**	37.2 (30.6, 44.1)	26.2 (20.0, 33.1)	15.2 (11.3, 19.7)	16.1 (12.2, 20.7)	19.9 (15.6, 24.8)	20.2 (16.0, 24.9)	22.3 (18.7, 26.2)	20.3 (16.8, 24.1)
**Meningitis**	1.2 (0.2, 3.8)	7.3 (4.1, 11.7)	0.9 (0.2, 2.8)	3.5 (1.8, 6.1)	4.2 (2.4, 6.7)	6.7 (4.4, 9.5)	1.2 (0.4, 2.6)	4.0 (2.6, 5.8)
**Diarrhea**	0.6 (0.0, 2.7)	0.6 (0.0, 2.7)	1.7 (0.6, 3.7)	0.3 (0.0, 1.5)	5.7 (3.7, 8.3)	0.5 (0.1, 1.6)	2.9 (1.7, 4.4)	2.3 (1.3, 3.7)
**Pneumonia**	18.9 (13.4, 25.4)	16.5 (11.7, 22.2)	30.9 (25.5, 36.6)	41.3 (35.6, 47.1)	11.6 (8.5, 15.2)	18.5 (13.9, 23.9)	19.9 (16.8, 23.2)	30.1 (26.5, 33.8)
**Sepsis**	25.0 (18.9, 31.8)	17.7 (12.1, 24.4)	23.3 (18.7, 28.2)	13.6 (10.0, 17.8)	37.4 (32.5, 42.5)	39.2 (33.8, 44.8)	31.5 (27.6, 35.6)	17.9 (14.9, 21.3)
**Other**	1.2 (0.2, 3.7)	3.7 (1.5, 7.1)	2.9 (1.4, 5.3)	0.0 (−, −)	2.6 (1.4, 4.5)	6.1 (4.2, 8.5)	4.8 (3.3, 6.5)	8.2 (6.1, 10.7)
**Preterm**	5.5 (2.7, 9.6)	17.1 (11.8, 23.4)	7.5 (4.8, 11.0)	17.6 (13.5, 22.2)	2.7 (0.6, 7.4)	2.2 (1.1, 3.9)	1.9 (1.0, 3.3)	3.8 (2.5, 5.6)
**Unspecified**	6.1 (3.1, 10.4)	11.0 (6.7, 16.6)	15.8 (11.9, 20.4)	7.6 (4.8, 11.1)	9.2 (6.7, 12.1)	6.0 (4.0, 8.3)	13.4 (10.5, 16.7)	11.9 (9.4, 14.7)
***Children***								
**Injury**	4.1 (2.6, 6.1)	3.6 (2.3, 5.4)	2.7 (1.3, 4.7)	2.7 (1.3, 4.7)	0.9 (0.4, 1.8)	0.8 (0.3, 1.6)	2.8 (2.1, 3.7)	2.8 (2.1, 3.7)
**AIDS**	3.3 (2.2, 4.7)	6.0 (4.0, 8.5)	0.6 (0.2, 1.2)	13.0 (10.5, 15.8)	2.8 (1.7, 4.3)	0.2 (0.0, 0.8)	0.6 (0.4, 1.1)	0.1 (0.0, 0.3)
**Malnutrition**	2.7 (1.6, 4.1)	8.0 (6.0, 10.5)	1.6 (0.8, 2.8)	7.8 (5.9, 10.1)	2.3 (0.7, 5.3)	0.4 (0.1, 1.2)	0.6 (0.3, 1.0)	4.1 (3.2, 5.1)
**Measles**	0.3 (0.1, 1.0)	0.8 (0.3, 1.7)	1.3 (0.6, 2.4)	0.8 (0.3, 1.7)	1.4 (0.6, 2.6)	3.0 (1.6, 5.0)	2.0 (1.4, 2.8)	4.4 (3.3, 5.7)
**Meningitis**	16.5 (13.9, 19.5)	16.4 (13.6, 19.5)	10.3 (8.2, 12.8)	4.3 (3.0, 6.1)	18.3 (15.1, 21.8)	34.1 (29.6, 38.7)	5.7 (4.6, 6.9)	9.0 (7.5, 10.6)
**Dysentery**	4.3 (2.8, 6.1)	3.6 (2.3, 5.4)	3.2 (2.1, 4.7)	2.7 (1.6, 4.1)	6.3 (4.5, 8.5)	0.1 (0.0, 0.5)	3.7 (2.9, 4.7)	3.4 (2.7, 4.4)
**Diarrhea**	21.9 (19.2, 24.7)	6.6 (4.8, 8.8)	18.4 (15.6, 21.5)	12.4 (9.9, 15.2)	19.5 (15.9, 23.4)	2.2 (1.2, 3.6)	22.3 (20.2, 24.5)	23.1 (20.9, 25.4)
**Pertussis**	1.3 (0.5, 2.4)	6.5 (4.6, 8.7)	1.0 (0.4, 2.0)	1.5 (0.7, 2.6)	0.3 (0.0, 1.4)	8.4 (5.7, 11.9)	0.6 (0.3, 1.0)	5.0 (3.9, 6.2)
**Pneumonia**	20.8 (17.5, 24.3)	16.9 (13.9, 20.1)	25.5 (22.3, 28.9)	29.2 (25.7, 32.9)	11.8 (8.9, 15.1)	16.1 (13.0, 19.6)	16.4 (14.6, 18.4)	19.6 (17.6, 21.8)
**Malaria**	19.1 (16.3, 22.1)	22.5 (19.3, 26.0)	24.7 (21.4, 28.2)	18.7 (15.8, 21.8)	28.9 (25.2, 32.9)	30.4 (26.1, 34.9)	36.3 (33.9, 38.8)	23.7 (21.4, 26.1)
**Other**	1.1 (0.5, 2.1)	5.8 (4.2, 7.9)	1.6 (0.8, 2.9)	1.9 (1.0, 3.1)	3.0 (1.6, 5.0)	1.9 (0.4, 5.0)	3.0 (2.2, 3.9)	2.2 (1.5, 3.1)
**Unspecified**	4.7 (3.3, 6.5)	3.3 (2.2, 4.8)	9.1 (7.0, 11.6)	5.0 (3.5, 6.9)	4.5 (3.0, 6.4)	2.4 (1.4, 3.9)	5.9 (4.8, 7.1)	2.6 (1.9, 3.4)

*EAVA* expert algorithm verbal autopsy, *PCVA* physician-coded verbal autopsy, *BA/BI* birth asphyxia/birth injury

^±^Not estimable

**Fig. 1:**
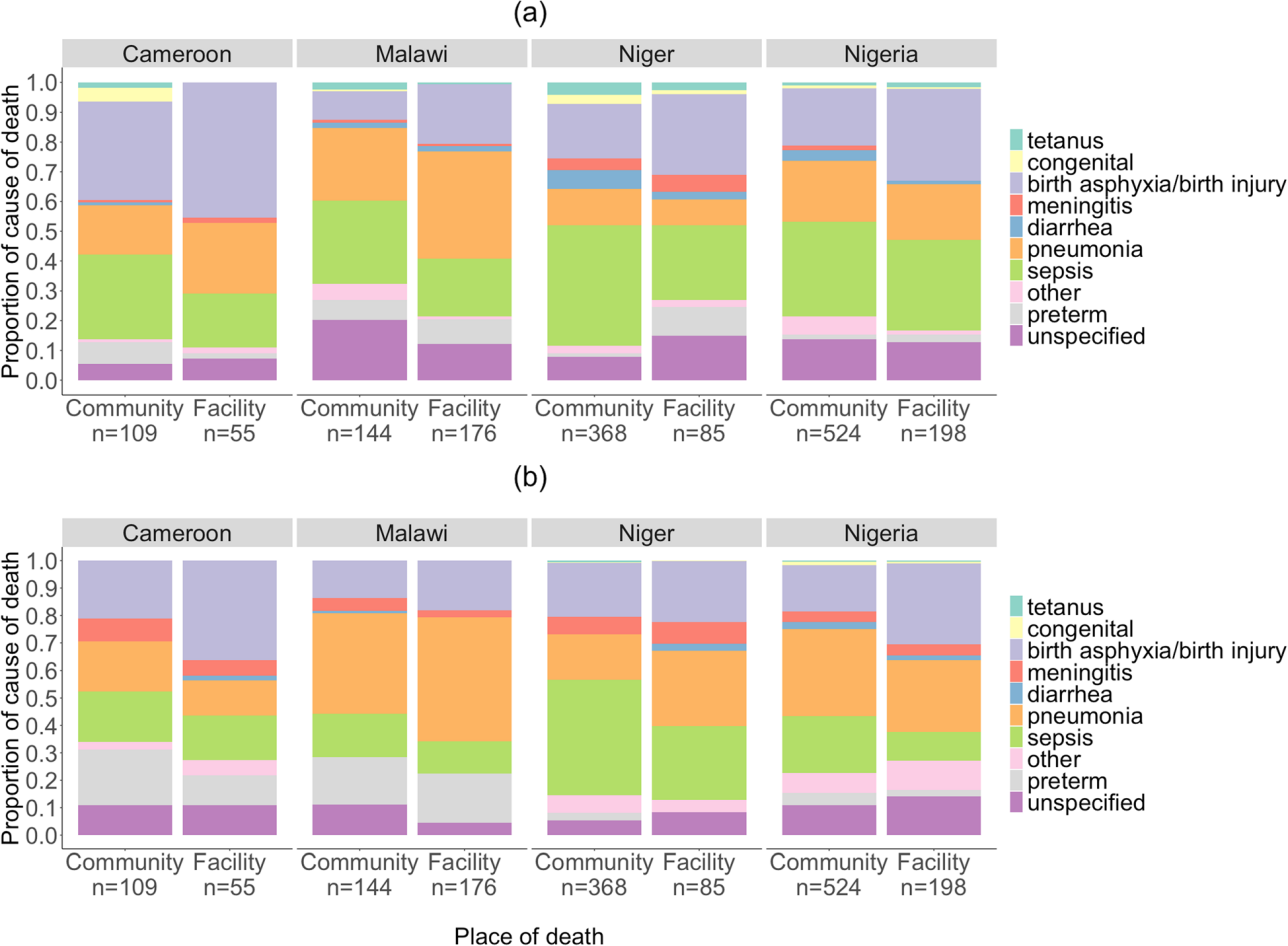
Verbal autopsy causes of neonatal deaths in communities and health facilities, Cameroon, Malawi, Niger, and Nigeria. **a** Expert algorithm neonatal causes of death. **b** Physician-coded neonatal causes of death

**Fig. 2:**
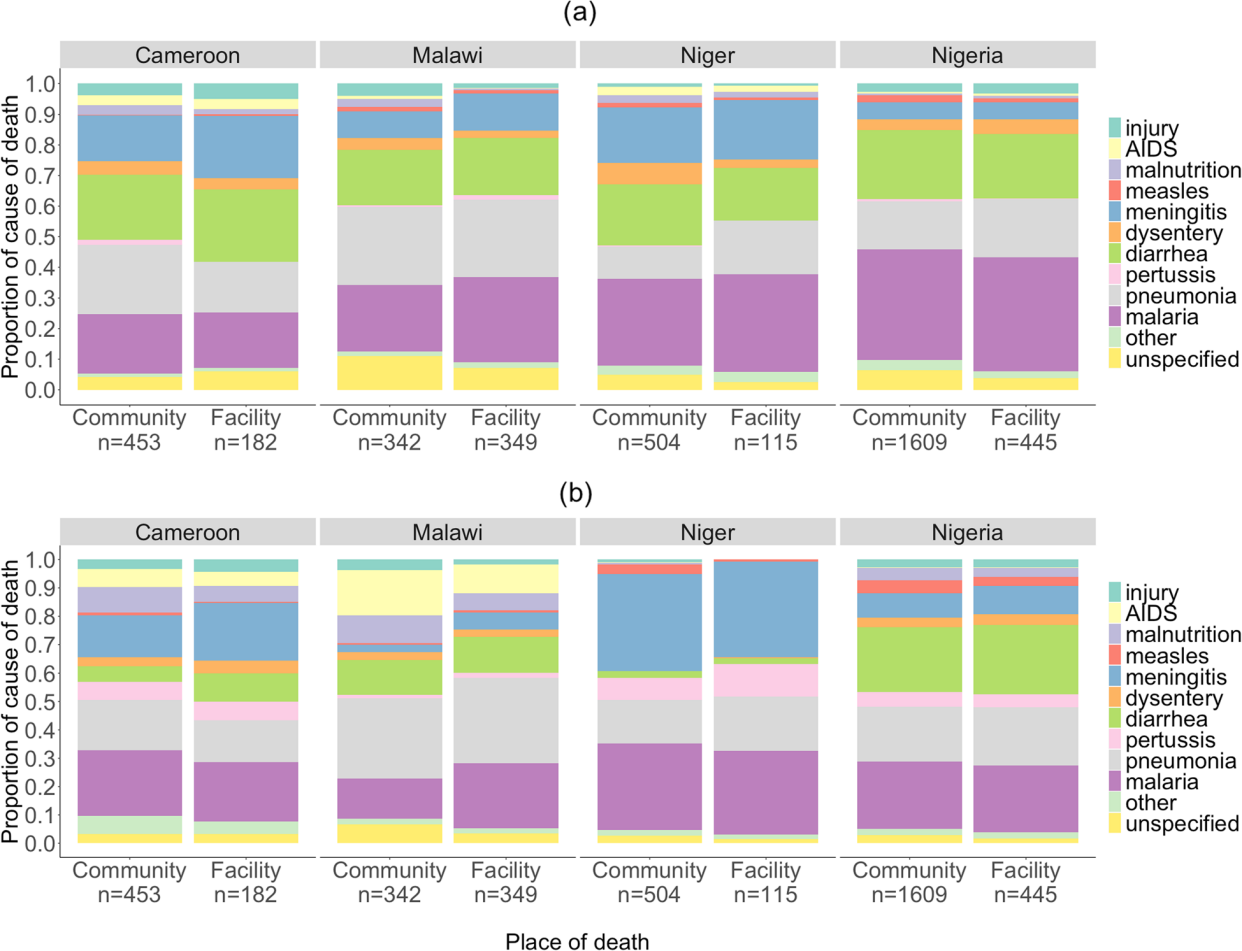
Verbal autopsy causes of child deaths in communities and health facilities, Cameroon, Malawi, Niger, and Nigeria. **a** Expert algorithm 1–59-month causes of death. **b** Physician-coded 1–59-month causes of death

Figures 1a, b and 2a, b show the EAVA and PCVA neonatal and 1–59-month community and health facility cause distributions for the four countries, and Tables 2 and 3 show the causes of death for which there were significant differences between the community and health facility proportions.

**Table 2:** Significantly different community and health facility causes of neonatal deaths in the study countries

Country	Analysis method	Cause of death	Community ***N*** deaths (%) %MF (95% CI)	Facility ***N*** deaths (%) %MF (95% CI)	p*
**Cameroon**			**109 (66.5%)**	**55 (33.5%)**	
	**EAVA**	**Non-significant**	–	–	–
	**PCVA**	**Birth asphyxia/birth injury**	21.1 (14.7, 28.6)	36.4 (24.0, 50.1)	0.023
**Malawi**			**144 (45.0%)**	**176 (55.0%)**	
	**EAVA**	**Birth asphyxia/birth injury**	9.4 (5.4, 14.9)	20.0 (14.0, 27.0)	0.011
		**Pneumonia**	24.5 (17.9, 32.0)	36.1 (28.4, 44.3)	0.027
		**Other**	5.3 (2.3, 10.2)	1.0 (0.2, 3.0)	0.015
		**Unspecified**	20.3 (13.9, 27.9)	12.2 (7.9, 17.6)	0.049
	**PCVA**	**Unspecified**	11.2 (6.4, 17.5)	4.6 (2.1, 8.4)	0.028
**Niger**			**368 (81.2%)**	**85 (18.8%)**	
	**EAVA**	**Sepsis**	40.3 (35.0, 45.7)	25.0 (15.5, 36.3)	0.019
		**Preterm**	1.2 (0.4, 2.5)	9.6 (0.8, 33.6)	0.011
	**PCVA**	**Diarrhea**	0.0 (−,^±^ −)	2.7 (0.4, 8.6)	0.031**
		**Sepsis**	42.1 (36.1, 48.2)	26.8 (16.7, 38.8)	0.026
**Nigeria**			**524 (72.6%)**	**198 (27.4%)**	
	**EAVA**	**Birth asphyxia/birth injury**	19.1 (15.2, 23.5)	30.8 (23.3, 39.0)	0.006
		**Other**	6.0 (4.1, 8.4)	1.4 (0.4, 3.5)	0.006
	**PCVA**	**Birth asphyxia/birth injury**	16.9 (13.2, 21.1)	29.4 (22.0, 37.7)	0.002
		**Sepsis**	20.7 (17.1, 24.8)	10.6 (6.0, 16.8)	0.007

*MF* mortality fraction, *EAVA* expert algorithm verbal autopsy, *PCVA* physician-coded verbal autopsy

*Significance determined by Rao-Scott chi-square

**Significance determined by Fisher’s exact test

^±^Not estimable

**Table 3:** Significantly different community and health facility causes of 1–59-month deaths in the study countries

Country	Analysis method	Cause of death	Community N deaths (%) %MF (95% CI)	Facility N deaths (%) %MF (95% CI)	p*
**Cameroon**			**453 (71.3%)**	**182 (28.7%)**	
	**EAVA**	**Non-significant**	–	–	–
	**PCVA**	**Diarrhea**	5.3 (3.4, 7.8)	9.9 (6.3, 14.4)	0.030
**Malawi**			**342 (49.5%)**	**349 (50.5%)**	
	**EAVA**	**Injury**	4.0 (1.9, 7.4)	1.4 (0.4, 3.4)	0.048
		**Severe malnutrition**	2.6 (1.2, 4.7)	0.6 (0.1, 1.9)	0.048
	**PCVA**	**AIDS**	15.9 (12.0, 20.4)	10.2 (7.2, 13.8)	0.034
		**Meningitis**	2.6 (1.3, 4.6)	6.0 (3.7, 9.0)	0.030
		**Malaria**	14.3 (10.8, 18.4)	22.9 (18.5, 27.8)	0.005
**Niger**			**504 (81.4%)**	**115 (18.6%)**	
	**EAVA**	**Non-significant**	–	–	–
	**PCVA**	**Non-significant**	–	–	–
**Nigeria**			**1610 (78.4%)**	**445 (21.6%)**	
	**EAVA**	**Unspecified**	6.5 (5.2, 7.8)	3.9 (2.2, 6.0)	0.044
	**PCVA**	**AIDS**	0.0 (0.0, 0.1)	0.3 (0.0, 1.4)	0.024

*MF* mortality fraction, *EAVA* expert algorithm verbal autopsy, *PCVA* physician-coded verbal autopsy

*Significance determined by Rao-Scott chi-square

For neonatal deaths, there were significant differences between the community and health facility proportions of birth injury/asphyxia by the EAVA or PCVA analysis method in three countries, of sepsis by one or both VA methods in two countries, and for preterm delivery, pneumonia, and diarrhea, each by one VA method in one country. Examining the findings by country, Malawi had the most causes with differences between the community and health facility proportions, with two of the four such causes being “other” and “unspecified.” Niger and Nigeria each had significant differences between the community and health facility proportions for three neonatal causes (one of these, in Nigeria, being “other”), and Cameroon had such for just one cause.

AIDS was the only 1–59-month cause of death for which there was a significant difference between the community and health facility proportions in more than one country (Nigeria and Malawi), and Malawi was the only country for which this was true for more than one cause. For all the neonatal and 1–59-month causes not shown in Tables 2 and 3, the community and health facility proportions were similar, with *p* values above 0.05 (Additional files 1a and b).

### Comparison of cause distributions

Table 4 displays the CSMF accuracy for the comparison of the overall EAVA and PCVA distributions of neonatal and 1–59-month causes of death that occurred in the community and in health facilities in each country. CSMF accuracy was generally high in these settings, indicating similarity between causes of community and health facility deaths. The largest differences between community and facility deaths were among neonates in Niger (0.74 for EAVA) and Malawi (0.76 for EAVA). The most similarity was among children in Nigeria (0.93 for EAVA and 0.95 for PCVA). CSMF accuracy also tended to be higher for deaths among children (average 0.90, range 0.85–0.95) than among neonates (average 0.80, range 0.74–0.87), indicating more similar cause distributions for children versus neonates.

**Table 4:** CSMF accuracy comparing the cause distributions of community and health facility deaths in the study countries

Country	Analysis method	Neonatal cause of death distribution, estimate (95% CI)	Child cause of death distribution, estimate (95% CI)
**Cameroon**	**EAVA**	0.77 (0.61, 0.86)	0.89 (0.78, 0.91)
**Cameroon**	**PCVA**	0.80 (0.61, 0.87)	0.87 (0.77, 0.90)
**Malawi**	**EAVA**	0.76 (0.64, 0.85)	0.87 (0.79, 0.91)
**Malawi**	**PCVA**	0.87 (0.75, 0.92)	0.85 (0.76, 0.88)
**Niger**	**EAVA**	0.74 (0.59, 0.84)	0.88 (0.71, 0.90)
**Niger**	**PCVA**	0.79 (0.63, 0.88)	0.92 (0.75, 0.93)
**Nigeria**	**EAVA**	0.87 (0.76, 0.90)	0.93 (0.86, 0.95)
**Nigeria**	**PCVA**	0.81 (0.70, 0.87)	0.95 (0.87, 0.95)

*CSMF* cause-specific mortality fraction, *EAVA* expert algorithm verbal autopsy, *PCVA* physician-coded verbal autopsy

CSMF accuracy also varied across countries. For neonates, accuracy was highest for EAVA in Nigeria (0.87) and for PCVA in Malawi (0.87), and for both EAVA and PCVA was lowest in Niger (0.74 and 0.79), while for children, CSMF accuracy both by EAVA and PCVA was highest in Nigeria (respectively, 0.93 and 0.95) and lowest in Malawi (0.87 and 0.85).

### Comparison of populations

Table 5 compares the distributions of demographic factors among the community and facility deaths. There were many significant differences in these factors, despite the similarities in their cause of death distributions. Among neonates, facility deaths were younger and more likely to have been born in a facility. Mothers of facility deaths were also wealthier and more educated than mothers of community deaths in all countries but Cameroon. Use of any antenatal care during the index child’s pregnancy, as an independent measure of access to health care, was greater for facility deaths in all countries but Niger.

**Table 5:** Demographics among community and health facility deaths in the study countries

Demographic factor	Cameroon			Malawi			Niger			Nigeria		
	Community deaths, ***N***	Facility deaths, ***N***	p*	Community deaths, ***N***	Facility deaths, ***N***	***p***	Community deaths, ***N***	Facility deaths, ***N***	***p***	Com-munity deaths, ***N***	Facility deaths, ***N***	***p***
***Neonates***	**109**	**55**		**144**	**176**		**368**	**85**		**524**	**198**	
**Age less than 7 days**	67%	80%	0.049	60%	78%	0.001	60%	90%	< 0.001	71%	82%	0.006
**Age 7–27 days**	33%	20%	0.049	40%	22%	0.001	40%	10%	< 0.001	29%	18%	0.006
**Mother’s education (none)**	3%	2%	0.690	18%	11%	0.073	91%	67%	0.004	63%	24%	< 0.001
**Mother’s education (none/primary)**	71%	64%	0.414	80%	64%	0.001	97%	90%	0.064	82%	49%	< 0.001
**Child male**	60%	56%	0.686	59%	55%	0.469	57%	62%	0.543	57%	58%	0.703
**Lowest wealth quintile**	19%	24%	0.584	31%	22%	0.080	30%	26%	0.576	34%	14%	< 0.001
**Highest wealth quintile**	17%	24%	0.375	13%	24%	0.012	13%	35%	0.014	11%	42%	< 0.001
**Born in facility**	20%	76%	< 0.001	45%	87%	< 0.001	18%	74%	< 0.001	17%	87%	< 0.001
**ANC (formal)**	72%	85%	0.040	90%	96%	0.032	73%	80%	0.312	51%	84%	< 0.001
***Children***	**453**	**182**		**342**	**349**		**504**	**115**		**1610**	**445**	
**Age 1–11 months**	42%	38%	0.374	56%	44%	0.006	45%	42%	0.747	34%	34%	0.847
**Age 12–23 months**	27%	26%	0.850	23%	24%	0.685	23%	16%	0.131	27%	29%	0.611
**Age 24–59 months**	31%	36%	0.240	21%	32%	0.004	32%	41%	0.205	39%	37%	0.505
**Mother’s education (none)**	5%	3%	0.268	26%	18%	0.011	89%	90%	0.922	68%	42%	< 0.001
**Mother’s education (none/primary)**	77%	72%	0.298	77%	77%	0.916	94%	94%	0.920	83%	67%	< 0.001
**Child male**	47%	53%	0.261	48%	48%	0.955	49%	49%	0.971	51%	52%	0.641
**Lowest wealth quintile**	30%	19%	0.006	29%	24%	0.111	27%	19%	0.055	22%	14%	< 0.001
**Highest wealth quintile**	19%	23%	0.313	18%	21%	0.218	15%	36%	0.003	13%	32%	< 0.001
**Born in facility**	25%	42%	< 0.001	70%	82%	0.001	23%	41%	0.011	20%	49%	< 0.001

*Significance determined by Rao-Scott chi-square

The balance in most demographic factors between community and facility deaths was similar for 1–59-month-olds to that for neonates. Child facility deaths in all four countries were more likely than were community deaths to have been born in a health facility. Likewise, mothers of child facility deaths in Malawi and Nigeria, but not in Cameroon and Niger, were less likely to have had no formal education, and in all countries but Niger, facility deaths were better off economically. However, in contrast to the age distributions for neonates in all four countries, in Malawi, child facility deaths were older than community deaths.

### Predicting population causes with facility deaths

Table 6 shows the CSMF accuracies for projections A, B, and E of the population distributions of neonatal and child causes of death compared to the observed population distributions shown in Table 1; projections C and D are included as well in Additional file 2. The simplest method to implement, substituting the observed causes of facility deaths for those in communities (projection A), had a CSMF accuracy for EAVA causes in neonates of 0.86 on average across the four countries (range 0.79–0.91), and for children an average of 0.93 (range 0.90–0.95). Average CSMF accuracies for neonatal (0.88) and child (0.93) PCVA causes were similar to those for EAVA causes, but with some variations across countries, the largest among neonates being for Malawi (EAVA 0.89 vs. PCVA 0.94) and Nigeria (EAVA 0.91 vs. PCVA 0.86) and among children for Niger (EAVA 0.90 vs. PCVA 0.94).

**Table 6:** Comparing population cause distributions estimated from facility deaths to observed population cause distributions in the study countries

***Age group*** Analysis method	Country	CSMF accuracy of population causes of death estimated from facility deaths compared to observed population causes				
		***N*** deaths	% facility deaths	Projection A* Estimate (95% CI)	Projection B** Estimate (95% CI)	Projection E*** Estimate (95% CI)
***Neonates***						
**EAVA**	**Cameroon**	**164**	**34%**	0.85 (0.76, 0.90)	0.84 (0.72, 0.89)	0.87 (0.75, 0.91)
	**Malawi**	**320**	**55%**	0.89 (0.84, 0.94)	0.91 (0.85, 0.94)	0.93 (0.85, 0.95)
	**Niger**	**453**	**19%**	0.79 (0.67, 0.86)	0.75 (0.59, 0.84)	0.84 (0.66, 0.88)
	**Nigeria**	**722**	**27%**	0.91 (0.83, 0.93)	0.92 (0.82, 0.93)	0.93 (0.79, 0.93)
	**Average**			0.86	0.86	0.89
**PCVA**	**Cameroon**	**164**	**34%**	0.87 (0.73, 0.90)	0.87 (0.74, 0.92)	0.89 (0.74, 0.92)
	**Malawi**	**320**	**55%**	0.94 (0.88, 0.96)	0.95 (0.89, 0.97)	0.94 (0.88, 0.96)
	**Niger**	**453**	**19%**	0.83 (0.70, 0.89)	0.87 (0.69, 0.91)	0.83 (0.67, 0.87)
	**Nigeria**	**722**	**27%**	0.86 (0.77, 0.90)	0.88 (0.76, 0.93)	0.85 (0.74, 0.90)
	**Average**			0.88	0.89	0.88
***Children***						
**EAVA**	**Cameroon**	**635**	**29%**	0.92 (0.85, 0.94)	0.91 (0.84, 0.94)	0.91 (0.85, 0.93)
	**Malawi**	**691**	**50%**	0.94 (0.90, 0.96)	0.95 (0.90, 0.96)	0.94 (0.89, 0.95)
	**Niger**	**619**	**19%**	0.90 (0.77, 0.92)	0.90 (0.40, 0.92)	0.92 (0.80, 0.93)
	**Nigeria**	**2055**	**22%**	0.95 (0.89, 0.96)	0.94 (0.88, 0.95)	0.95 (0.89, 0.96)
	**Average**			0.93	0.93	0.93
**PCVA**	**Cameroon**	**635**	**29%**	0.91 (0.83, 0.93)	0.90 (0.83, 0.93)	0.91 (0.83, 0.93)
	**Malawi**	**691**	**50%**	0.92 (0.88, 0.94)	0.92 (0.88, 0.94)	0.92 (0.87, 0.94)
	**Niger**	**619**	**19%**	0.94 (0.81, 0.94)	0.92 (0.79, 0.94)	0.92 (0.81, 0.94)
	**Nigeria**	**2055**	**22%**	0.96 (0.89, 0.96)	0.97 (0.89, 0.97)	0.95 (0.89, 0.96)
	**Average**			0.93	0.93	0.93

Observed and estimated population cause distributions are from verbal autopsies

*CSMF* cause-specific mortality fraction, *EAVA* expert algorithm verbal autopsy, *PCVA* physician-coded verbal autopsy

*Population causes estimated by substituting observed causes from facility deaths for community deaths

**Population causes estimated by predicting community causes with multinomial logistic regression of facility deaths based on age at death, mother’s education, and receiving any ANC (only for neonates, yes/no)

***Population causes estimated with random forest, using same predictors as multinomial projection B, plus birthplace

In general, adjusting for demographic factors did not yield substantial improvement over the simple unadjusted projection A. Average CSMF accuracy for the multinomial logistic regression (projection B) for EAVA causes was identical to that for projection A in neonates (0.86) and children (0.93), and for PCVA causes was identical in children (0.93) and nearly so in neonates (0.89 vs. 0.88). CSMF accuracies for the two projections within each country were also very similar. Average and within-country CSMF accuracies for RF projection E of EAVA and PCVA causes in children and PCVA causes in neonates also were very similar to those for projection A. Only the projection E average and within-country CSMF accuracies for EAVA causes in neonates showed consistent increases over those for projection A. CSMF accuracies for RF projection C (shown in Additional file 2) were similar to those for RF projection E.

**Fig. 3:**
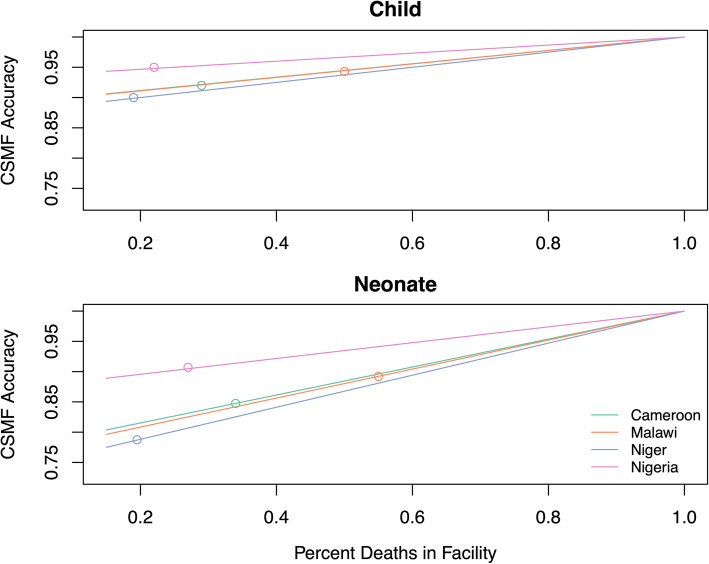
Relationship between percent of deaths in health facilities and predicted EAVA population cause-specific mortality fraction accuracy. Dots indicate the measured CSMF accuracy at the level of health facility deaths in each country

Figure 3 depicts the hypothetical relationship between the percent of deaths occurring in facilities and the CSMF accuracy of facility causes for the population cause distribution, for neonatal and 1–59-month-old deaths in each of the study countries. It can be seen that CSMF accuracy increases as the proportion of all deaths that occur in facilities increases.

## Discussion

Verbal autopsy is the main source of data on causes of neonatal and child death in countries with weak civil registration and vital statistics systems, including over 80 countries with the highest burden of under-five mortality [31]. The critical nature of the accuracy and representativeness of VA data, whether for local or high level modeling purposes, is apparent. Our analysis found few differences in the fraction of specific causes between health facility and community, with variations from one to four of 10 examined neonatal causes and from one to three of 12 child causes by either VA analysis method across the four VASA studies. While there were large within-country differences between the EAVA and PCVA population proportions for some causes, this was not unexpected, as it is common for different VA analysis methods to yield different cause-specific proportions. What matters is that the within-country, within-method facility and community cause proportions were similar to each other. Since for each method, all the deaths were analyzed in the same way, this should not have been a factor in the comparison of facility and community causes. We also found high levels of CSMF accuracy between health facility and community deaths, generally exceeding those of the InSilicoVA method comparing estimated versus high-quality causes of death across (0.70) and within (0.85) study sites [15], as well as the Tariff 2.0 VA method comparing estimated versus high-quality causes of death for neonates (0.83) and 1–59-month-olds (0.78) [13].

This means that a health program manager utilizing neonatal and child medically determined causes of death in health facilities to estimate the population neonatal and child CSMFs could expect to achieve reasonable accuracy, given the following caveats: first, that the sample of facilities is reasonably representative of those in the population. Prior studies that found or presumed that facility deaths would find non-representative causes have examined or referenced hospital deaths, without including lower level facilities [1, 2]. The higher the facility level, especially secondary and tertiary hospitals with greater selectivity of patients, the less might one expect the causes of death to be representative of all deaths. Surveillance at 16 tertiary care hospitals throughout India found that infection was the third leading cause of neonatal death, after prematurity and birth asphyxia [32], whereas at a sub-district hospital in Haryana during the same time frame, there were as many neonatal deaths due to septicemia as from prematurity and birth asphyxia combined [33], similar to the mortality pattern found in a community study in rural Maharashtra [34]. The deaths in our study were identified by representative household surveys and so included deaths from wherever they occurred, be that in the community or a health facility of any level. We were not able to identify other studies that have compared head-to-head the causes of under-5 mortality in representative samples of community and facility deaths. It would be worthwhile to conduct analyses like those reported here in additional countries and regions to further examine this issue. There are also several published studies of child mortality from sample registration systems that could be re-explored to determine if they recorded place of death.

Second, the EAVA and PCVA methods utilized by our study assessed only the most prevalent causes of neonatal and 1–59-month-old deaths in low-income countries. Further work would be needed to determine if there is a similar correlation of hospital vs. community deaths for less frequent causes.

We attempted to improve on crude CSMF accuracy by adjusting for factors expected to influence health care access and utilization and therefore place of death, although how factors would be related to cause and place of death together is less intuitive. None of the adjusted projections of child causes of death increased CSMF accuracy substantially over the unadjusted projection.

Prior work suggests there are few causes of death for which health care is expected to be utilized more or less than for others. Illnesses characterized by convulsions, such as tetanus, meningitis and, in Tanzania, cerebral malaria [35], may be interpreted as having a spiritual cause and so not amenable to health care. However, this belief can vary, so is not easily predictable; in Mandiana, Guinea, mothers were more likely to seek health care for their children with convulsions [36]. Among the four VASA studies, only in Malawi were meningitis and malaria deaths proportionally higher in facilities than the community (Table 3). Meningitis proportions were much greater in Niger and Cameroon, and malaria somewhat higher, but both were equally distributed between facilities and community (Additional file 1b). Various explanations might be posited, such as past experience with the health system and setting-specific responses to particular symptoms. Access to child health care is an obvious possibility, but the DHS does not include a good measure of access for severe illness.

AIDS deaths in Malawi were more frequent in the community than facilities. Several HIV/AIDS program factors might explain this discrepancy. During the period examined by the VASA study, antiretroviral therapy (ART) of HIV in Malawi was based on WHO guidelines requiring a clinical stage 3 or 4 condition or a low CD4 count [37]; few HIV-positive women were receiving ART, leading to high levels of mother-to-child transmission; and there were program implementation challenges including long delays before children’s PCR results confirming HIV infection became available, a lack of pediatric ARV formulations, and unsynchronized mother-child clinic visits. In this environment, many HIV-infected patients were developing incurable complications that were managed in the community with palliative care. Careseeking may also have been affected by stigma and discrimination against persons with HIV/AIDS, issues being tackled with strengthened interventions since the years of the VASA study [38]. In Nigeria, AIDS deaths were higher in facilities, but the proportions were extremely low. Severe malnutrition deaths in Malawi also were higher in the community than in facilities. Ministry of Health guidelines emphasize community management of acute malnutrition [39], and mothers of children with complications may resist multiple health facility admissions due to the economic impact.

One might hypothesize that deaths from perinatal conditions that kill quickly, such as birth asphyxia and prematurity, are more likely to occur in health facilities in settings where many births occur in facilities and/or there is good health careseeking for maternal delivery complications. And, indeed, projections including birthplace outperformed those that did not, suggesting that birthplace may be related to place of neonatal death and cause of death. This was evident in our VASA data, with significantly more neonatal facility decedents born in facilities than in the community in all four countries and more facility than community deaths from birth asphyxia and prematurity in, respectively, three and one countries. In contrast, sepsis caused more community than facility deaths in two countries. These findings paralleled those of the India National Neonatal Perinatal Database, which found that prematurity and birth asphyxia predominated as causes of neonatal death in cases born in a tertiary hospital, whereas septicemia was the most common cause in neonates admitted from nursing homes and small hospitals [40].

While we were not able to identify any studies that compared facility and community causes of death directly, there is prior research relating to the prediction of community or population causes from facility deaths. Whiting et al. applied CSMFs estimated from facility deaths to counts of nearby sentinel vital registration (SVR) deaths, similar to our unadjusted projection [16], but tested their method comparing facility causes with medical certification to all VA-assessed SVR deaths. Other methods have required external sources of cause-specific mortality in communities. Murray et al. estimated the proportion of cause-specific mortality occurring in facilities from deaths reported in a national CRVS system to predict the population CSMF, specific to age and sex [17], a method extended by Williams et al., who used logistic regression to estimate the probability by cause of dying in a health facility based on a broad range of covariates including age, sex, and extensive demographic information, and reweighted the CSMFs with predicted probabilities to estimate the population cause distribution [18]. We have extended this work to examine how representative facility data could be used in a practical situation without relying on cause of death information from other sources. Figure 3 shows to what extent our unadjusted method might be influenced by varying levels of facility death, suggesting its superior performance over community VA assessment of child deaths in all settings and of neonatal deaths in settings with 40% or more facility deaths. We have also examined the utility of more sophisticated cause projections based on demographic factors, which we found only marginally improved on the simpler estimate.

The implication of our findings is that just as we have done with VA causes, the medical causes of neonatal and child deaths from a representative sample of health facilities can be used to estimate the population distribution of the causes. Further research on how to select a representative sample of health facilities, improve medical certification, ICD coding, and reporting of causes of death is needed to support this effort.

### Limitations

We did not have data on medical causes of death from health facilities and communities to test our method on the type of data that we anticipate health program managers using the method would have access to. However, it would be impractical to have medical data on the causes of community deaths to test the method, for the very reason that VA is the main method used to estimate causes of community deaths in developing countries. This may become more practical in the near future, with the advent of new diagnostic technologies such as minimally invasive tissue sampling (MITS) being conducted on community deaths, although these are unlikely to ever be nationally representative.

The VASA studies were context-specific in that causes of death and their distributions across health facilities, communities, countries, and regions may vary with time. This concern is lessened by our having examined deaths that occurred over several years both in West and East Africa, but still remains.

## Conclusions

The verbal autopsy-based causes of facility and community deaths of neonates and 1–59-month-olds were found to be similar to each other in the four African countries. These findings suggest that population causes of neonatal and child deaths in developing country settings can be projected from a representative sample of facilities where children die. This method should be tested with more recent deaths in additional countries and regions. If good agreement is found between facility and community deaths generally or in some settings, research should be conducted in selecting a representative sample of health facilities and strengthening medical certification so that VA will no longer be necessary.

## Supplementary information

**Additional file 1.** a. Community and health facility causes of neonatal deaths in four sub-Saharan Africa countries. b. Community and health facility causes of 1–59-month deaths in four sub-Saharan Africa countries.

**Additional file 2.** All projections of CSMF accuracy comparing population cause distributions estimated with verbal autopsies of facility deaths to observed population cause distributions from verbal autopsies of community and facility deaths, in four sub-Saharan Africa countries.

## Data Availability

The datasets analyzed during the current study are available at the following URL on the Johns Hopkins Bloomberg School of Public Health, Institute for International Program’s Improve Project website: https://www.jhsph.edu/research/centers-and-institutes/institute-for-international-programs/current-projects/improving-measurement-and-program-design/community-facility-COD-4countries.zip.

## Abbreviations

ART: Antiretroviral therapy
CI: Confidence interval
CRVS: Civil registration and vital statistics
CSMF: Cause-specific mortality fraction
DHS: Demographic and Health Survey
EAVA: Expert algorithm verbal autopsy
MITS: Minimally invasive tissue sampling
MR: Mortality fraction
MLR: Multinomial logistic regression
PCVA: Physician-coded verbal autopsy
RF: Random forests
SVR: Sentinel vital registration
VA: Verbal autopsy
VASA: Verbal and social autopsy
